# Validation of the short forms of the Pelvic Floor Distress Inventory (PFDI-20), Pelvic Floor Impact Questionnaire (PFIQ-7), and Pelvic Organ Prolapse/Urinary Incontinence Sexual Questionnaire (PISQ-12) in Finnish

**DOI:** 10.1186/s12955-017-0648-2

**Published:** 2017-05-02

**Authors:** Nina Kristiina Mattsson, Kari Nieminen, Anna-Mari Heikkinen, Jyrki Jalkanen, Sari Koivurova, Marja-Liisa Eloranta, Pia Suvitie, Anna-Maija Tolppanen

**Affiliations:** 1 Department of Obstetrics and Gynecology, Kanta-Häme Central Hospital, Hämeenlinna, Finland; 2 Department of Obstetrics and Gynecology, Tampere University Hospital, Tampere, Finland; 3 Terveystalo, Kuopio, Finland; 4 Department of Obstetrics and Gynecology, Central Finland Hospital District, Jyväskylä, Finland; 5 Department of Obstetrics and Gynecology, Oulu University Hospital, Oulu, Finland; 6 Department of Obstetrics and Gynecology, Kuopio University Hospital, Kuopio, Finland; 7 Department of Obstetrics and Gynecology, Turku University Hospital and University of Turku, Turku, Finland; 8 Research Centre for Comparative Effectiveness and Patient Safety (RECEPS) and School of Pharmacy, University of Eastern Finland, Kuopio, Finland

**Keywords:** Pelvic organ prolapse, Symptom questionnaire, Validation, Psychometric evaluation, Reliability, Health related quality of life

## Abstract

**Background:**

Although several validated generic health-related quality of life instruments exist, disease-specific instruments are important as they are often more sensitive to changes in symptom severity. It is essential to validate the instruments in a new population and language before their use. The objective of the study was to translate into Finnish the short forms of three condition-specific questionnaires (PFDI-20, PFIQ-7 and PISQ-12) and to evaluate their psychometric properties in Finnish women with symptomatic pelvic organ prolapse.

**Methods:**

A multistep translation method was used followed by an evaluation of validity and reliability in prolapse patients. Convergent and discriminant validity, internal consistency and reliability via test-retest were calculated.

**Results:**

Sixty-three patients waiting for prolapse surgery filled the three questionnaires within two weeks. Response rate for each item was high in PFDI-20 and PISQ-12 (99.8 and 98.9% respectively). For PFIQ-7 response rate was only 60%. In PFIQ-7, six respondents (9.5%) reached the minimum value of zero showing floor effect. None of the instruments had ceiling effect. Based on the item-total correlations both PFIQ-7 and PFDI-20 had acceptable convergent validity, while the convergent validity of PISQ-12 was lower, r = 0.138–0.711. However, in this instrument only three questions (questions 6, 10 and 11) had r < 0.3 while others had r ≥ 0.380. In the test-retest analysis all the three instruments showed good reliability (ICC 0.75–0.92). Similarly, the internal consistency of the instruments, measured by Cronbach’s α, was good (range 0.69–0.96) indicating high homogeneity.

**Conclusions:**

Finnish validated translation of the PFDI-20 and PISQ-12 have acceptable psychometric properties and can be used for both research purposes and clinical evaluation of pelvic organ prolapse symptoms. The Finnish version of PFIQ-7 displayed low response rate and some evidence of a floor effect, and thus its use is not recommended in its current form.

**Electronic supplementary material:**

The online version of this article (doi:10.1186/s12955-017-0648-2) contains supplementary material, which is available to authorized users.

## Background

Pelvic floor disorders (PFD) include variable symptoms such as urinary incontinence, feeling of a vaginal bulge, fecal incontinence, and other sensory and emptying abnormalities of the lower urinary and gastrointestinal tracts. The prevalene of women reporting at least one pelvic floor disorder is 23%, which proportion increases with age [1]. These symptoms can have a significant impact on the quality of life and they may cause problems in sexual life [2]. The prevalence of symptomatic pelvic organ prolapse (POP) is estimated to be 3–6% of adult women and up to 50% when based upon vaginal examination [1, 3]. It is necessary to consider not only the underlying anatomical disorder but also women's overall pelvic function and their health-related quality of life when making treatment decisions [3]. For this purpose, condition-specific quality-of-life instruments were developed and published in English 2001 [4, 5]. The Pelvic Floor Distress Inventory (PFDI), the Pelvic Floor Impact Questionnaire (PFIQ), and the Prolapse/Urinary Incontinence Sexual Questionnaire (PISQ) have shown to be psychometrically valid and reliable instruments for measuring the extent to which pelvic floor disorders affect quality of life [4, 5]. PFDI investigates the range of POP symptoms and the inconvenience they cause, while PFIQ covers the impact of POP on daily life. PISQ investigates the sexual function of heterosexual women suffering from POP and/or urinary incontinence. The short versions of these three questionnaires have also been validated [6, 7]. PFDI-20 consists of three separate scales: Pelvic Organ Prolapse Distress Inventory (POPDI) of six questions about the inconvenience of the prolapse, Colorectal-Anal Distress Inventory (CRADI) with eight questions concerning difficulties of defecation, and the Urinary Distress Inventory (UDI) with six questions about difficulties in urination. Similarly, the PFIQ-7 consists of three scales, each of them containing seven questions: the Pelvic Organ Prolapse Impact Questionnaire (POPIQ), the Colorectal-Anal Impact Questionnaire (CRAIQ) and the Urinary Impact Questionnaire (UIQ). The short version of PISQ contains 12 questions about sexual activity, satisfaction and problems caused by POP or urinary incontinence.

PFDI-20, PFIQ-7 and PISQ-12 are widely used and they help investigators to evaluate the efficacy of a particular therapy for POP or to compare symptom severity between patients or groups. These disease-specific instruments have been translated and validated in several different countries and in more than ten languages [8–20].

Validated tools for measuring the severity of discomfort of pelvic prolapse and assessing the effectiveness of therapy are not currently available in Finnish. The aim of this study was to translate PFDI-20, PFIQ-7 and PISQ-12 into Finnish and validate these translations among women with symptomatic POP.

## Methods

For the translation process, a group of seven key in-country persons (authors NM, KN, A-MH, JJ, SK, M-LE, PS) were recruited among the board of Finnish Society of Gynecological Surgery. Translation permissions were obtained from the developers of the instruments, Dr. Barber [4] and Dr. Rogers [5]. The translation of the forms was conducted by multistep translation method [21]. Four forward translations of PFDI-20, PFIQ-7 and PISQ were done, two by independent professional translators with experience of translating patient-reported outcome (PRO) measures, and two by PhD gynecologist experienced in urogynecology. The four translations were tested on a group of four lay persons. Two of the lay persons were urogynecological nurses and two native Finnish-speaking nonprofessionals, one of whom was bilingual (Finnish-English). Each lay person picked the best translation alternatives of the questions or proposed their own alternative wording. One gynecologist (NM) compared lay persons´ interpretation and made the final review of the translation. A professional medical translator performed back-translations that were compared to the original questionnaires. The final versions of the translated instruments were approved by the group of key-in country persons (Additional files 1, 2 and 3).

A test-retest analysis was conducted among 63 native Finnish-speaking female patients waiting for surgery for symptomatic POP. The women were recruited from four hospitals: Turku University Hospital, Kuopio University Hospital, Oulu University Hospital and Kanta-Häme Central Hospital. The first three are tertiary university hospitals and the last one is a secondary hospital, all performing urogynecological surgery. The hospitals represent different areas of Finland: western, eastern, northern and southern part of Finland, respectively.

Postal questionnaires including two pre-stamped envelopes were sent to the patients waiting for prolapse treatment. The patients were asked first to fill and return the test questionnaires and then, after 2 weeks, to fill out and return the re-test questionnaires. The questionnaires were paired by a code number and analysed anonymously. The participants gave their informed consent by returning the written questionnaires. The study was approved by the Ethical committee of University of Eastern Finland (2014/5), and it followed the ethical standards of the Helsinki Declaration [22].

### Statistical and data analysis

The PFIQ-7, PFDI-20 and PISQ-12 questionnaires and the subscales of PFIQ-7 and PFDI-20 were tested for construct validity and reliability. The average scores in each scale were reported as means and standard deviations, as well as medians and interquartile range due to the skewed distribution of the data. Convergent and discriminant validity were investigated with Spearman’s rank order correlation and the corrected item-total correlations. Corrected item-total correlations ≥0.3 can be considered as evidence on convergent validity [23]. In addition, response rate, floor and ceiling effects, (i.e., persons obtaining minimum and maximum scores, respectively) were calculated. Overall response rate was defined as the proportion of the patients that returned the two questionnaires in two weeks. Item response rate was defined as the proportion of answered questions in each questionnaire. Reliability was assessed by test-retest analysis and intra-class correlation coefficient (ICC), while the internal consistency was measured with Cronbach’s α. Cronbach’s α was calculated separately for persons with missing data and those who completed all questions in the subscale forms. α-values below 0.7 indicate too high heterogeneity, while values above 0.9 indicate too high similarity between items [24]. Thus, the preferred range of α is between 0.7 and 0.9.

Statistical analyses were conducted with Stata 14.0 (Stata Corporation, College Station TX, USA) and IBM SPSS 21.0 (Chicago IL, USA).

## Results

**Fig. 1:**
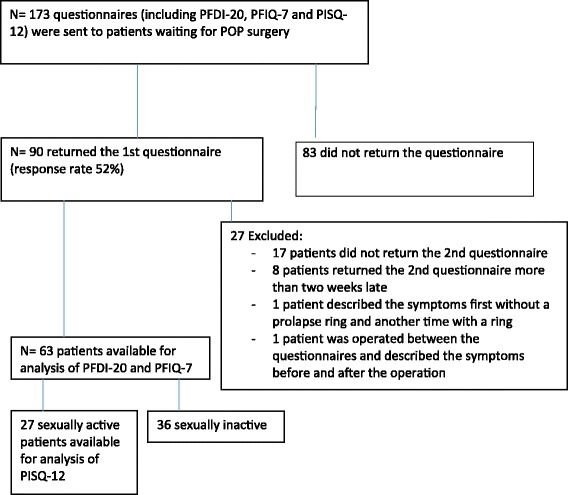
Flowchart: Inclusion of patient group

The formation of the study population is shown in Fig. 1. The final sample consisted of 63 women who returned both questionnaires. Twenty-seven of the 63 (42%) patients who returned both questionnaires were sexually active and completed the PISQ-12. The mean age of the patients was 64.1 years (median 64, range 25–86 years).

The item response rates were 99.8% for PFDI-20, 60.0% for PFIQ-7 and 98.9% for PISQ-12. In PFDI-20 factor scores without any imputations could be calculated in 96.8% cases for POPDI-6, 98.4% in cases for CRADI-8 and 100% in cases for UDI-6 (Table 1). For PFIQ-7, factor scores that could be calculated were 82.5% of cases for UIQ -7, 77.8% of cases for CRAIQ-7 and 79.4% of cases for POPIQ-7.

**Table 1:** Results from the analyses for reliability (test–retest and internal consistency), for each instrument and subscale

Questionnaire	Test			Retest			ICC (95% CI) P
	n (missing values)	Mean (SD), median (IQR)	Cronbach’s α (^a^)	n (missing values)	Mean (SD), median (IQR)	Cronbach’s α (^a^)	
PFIQ-7	63 (11)	14.97 (14.42) 9 (4–22)	0.96 (0.96)	63 (9)	13.97 (15.07) 7 (4–21)	0.94 (0.93)	0.75 (0.62–0.84) <0.001
UIQ-7	63 (5)	6.03 (5.77) 5 (1–9)	0.92 (0.92)	63 (6)	5.49 (5.67) 4 (1–9)	0.93 (0.93))	0.82 (0.72–0.88) <0.001
CRAIQ-7	63 (9)	3.83 (4.83) 2 (0–7)	0.91 (0.91)	63 (7)	3.98 (5.93) 1 (0–6)	0.77 (0.77)	0.67 (0.51–0.79) <0.001
POPIQ-7	63 (5)	5.11 (5.23) 3 (1–8)	0.92 (0.92)	63 (8)	4.89 (5.44) 3 (0–7)	0.93 (0.93)	0.72 (0.57–0.82) <0.001
PFDI-20	63 (3)	105.46 (55.62) 93.75 (70.83–146.88)	0.88 (0.88)	63 (0)	105.52 (58.07) 92.71 (66.67–158.33)	0.89 (0.89)	0.92 (0.88–0.95) <0.001
POPDI-6	63 (2)	40.15 (21.25) 37.50 (25.00–50.00)	0.73 (0.74)	63 (0)	42.86 (23.44) 37.50 (25.00–62.50)	0.78 (0.78)	0.83 (0.73–0.89) <0.001
CRADI-8	63 (1)	29.07 (21.78) 25.00 (9.38–46.88)	0.80 (0.80)	63 (0)	28.47 (22.29) 25.00 (9.38–40.63)	0.83 (0.83)	0.90 (0.84–0.94) <0.001
UDI-6	63 (0)	34.19 (21.78) 35.00 (29.00–40.00)	0.71 (0.71)	63 (0)	34.19 (21.78) 33.33 (16.67–50.00)	0.69 (0.69)	0.89 (0.83–0.93) <0.001
PISQ-12	27 (2)	34.89 (6.32) 35 (29–40)	0.84 (0.84)	27 (3)	31.85 (6.39) 33.00 (26.00–37.00)	0.82 (0.84)	0.87 (0.73–0.94) <0.001

^a^Computed only for those with complete answers on all questions of the subscale

Floor or ceiling effects were not observed with PFDI-20 or PISQ-12 instruments. There was little evidence of floor effect with subscales of PFIQ-7 (15–17% responders with minimum value), but no significant floor effect was observed with the summary scale, with four respondents (7%) having the minimum value of zero (Table 2). Ceiling effects were not observed.

**Table 2:** Floor and ceiling effects of baseline scores

Questionnaire (scores min-max)	Factor scores calculated (*n*)	Floor, *n* (%)	Ceiling, *n* (%)
PFDI-20 (0–300)	63	0 (0)	0 (0)
POPDI-6 (0–100)	63	0 (0)	1 (1.6)
CRADI-8 (0–100)	63	5 (8)	0 (0)
UDI-6 (0–100)	63	5 (8)	1 (1.6)
PFIQ-7 (0–300)	58	4 (7)	0 (0)
POPIQ-7 (0–100)	58	10 (17)	0 (0)
CRAIQ-7 (0–100)	59	18 (31)	0 (0)
UIQ-7 (0–100)	59	9 (15)	2 (3.4)
PISQ-12 (0–48)	27	0 (0)	0 (0)

Based on the item-total correlations, both PFIQ-7 and PFDI-20 had acceptable convergent validity (Additional file 4: Table S1 and Additional file 5: Table S2). The correlations were *r* = 0.601–0.878 for UIQ-7, *r* = 0.568–0.907 for CRAIQ- 7, *r* = 0.643–0.853 for POPIQ-7 and *r* = 0.513–0.865 for the total PFIQ-7. Lower item–total correlations were observed with PFDI-20 (*r* = 0.309-0.579 for POPDI-6, *r* = 0.371-0.486 for UDI-6, 0.335–0.611 for CRADI and *r* = 0.309-0.639 for the PFDI-20 total score). The lowest convergent validity was observed with PISQ-12, *r* = 0.138-0.711 (Additional file 6: Table S3). However, in this instrument only three questions (questions 6, 10 and 11) had *r* < 0.3, while others had *r* ≥ 0.380.

Convergent validity was analyzed by correlation between the three instruments (Table 3). Correlation between PFDI-20 and PFIQ-7 was 0.743, and ranged between 0.492 and 0.929, including the subscales. In both of these questionnaires, the total score correlated well with their respective subscales. PISQ-12 was negatively correlated with PFDI-20 and PFIQ-7 total scores and subscales (*r* = -0.327 to -0.616). Based on the strenght and direction of the item-total correlations, both PFIQ-7 and PFDI-20 had acceptable convergent validity (Additional file 4: Table S1 and Additional file 5: Table S2).

**Table 3:** Results from the analysis of convergent validity, i.e. correlation between the three questionnaires (including subscales). Data are given as r (P)

Questionnaire	PFIQ-7	UIQ-7	CRAIQ-7	POPIQ-7	PFDI-20	POPDI-6	CRADI-8	UDI-6
UIQ-7	0.929 (<0.001)							
CRAIQ-7	0.756 (<0.001)	0.621 (<0.001)						
POPIQ-7	0.847 (<0.001)	0.702 (<0.001)	0.522 (<0.001)					
PFDI-20	0.743 (<0.001)	0.683 (<0.001)	0.688 (<0.001)	0.565 (<0.001)				
POPDI-6	0.565 (<0.001)	0.497 (<0.001)	0.526 (<0.001)	0.459 (<0.001)	0.861 (<0.001)			
CRADI-8	0.623 (<0.001)	0.538 (<0.001)	0.739 (<0.001)	0.406 (0.001)	0.821 (<0.001)	0.572 (<0.001)		
UDI-6	0.691 (<0.001)	0.708 (<0.001)	0.492 (<0.001)	0.538 (<0.001)	0.841 (<0.001)	0.624 (<0.001)	0.549 (<0.001)	
PISQ-12	-0.511 (0.006)	-0.506 (0.007)	-0.432 (0.025)	-0.339 (0.084)	-0.616 (0.001)	-0.640 (<0.001)	-0.496 (0.009)	-0.327 (0.096)

In the test-retest analysis, all the three instruments showed good reliability (Table 1). Intra-class correlations were strong, varying from 0.75 (PFIQ-7) to 0.92 (PFDI-20). All ICCs were statistically significant (p < 0.001). Similarly, the internal consistency of the instruments, measured by Cronbach’s α, was between 0.69-0.89 for PISQ-12 and PFDI-20 and its subscales. α-values for baseline PFIQ-7 and its subscales were 0.91-0.96, indicating high homogeneity.

## Discussion

Pelvic organ prolapse itself, its treatment and complications related to it (for example de novo dyspareunia or vaginal mesh exposure following surgery) may have a significant effect on the patient’s quality of life. Hence, it is essential to measure the symptoms and HRQOL related to POP with validated instruments, both in clinical practice and research settings. PFDI-20, PISQ-12 and PFIQ-7 have proven to be valid and reliable instruments for measuring symptom inconvenience caused by pelvic organ prolapse and the health-related quality of life [6, 7]. Until now, their Finnish translations have not been validated. In the present study we have translated these questionnaires in Finnish and assessed the reliability and validity of these Finnish versions among women suffering from symptomatic pelvic organ prolapse in the present study.

The item response rates for PFDI-20 and PISQ-12 were high (99.8 and 98.9%, respectively), whereas the response rate for PFIQ-7 was only 60%. Ceiling effects were not observed. Floor effect was observed with all three subscales of PFIQ-7, but it was less evident with the summary scales. Cronbach’s α of PFIQ was 0.94 and 0.96 indicating that some of the items may be too similar. PFIQ also had the lowest ICC of 0.75, while the internal consistency of PFDI-20 and PISQ-12 was better (0.92 and 0.87, respectively). In addition, there was no evidence of too high homogeneity or heterogeneity of individual items in these scales, as indicated by Cronbach’s α.

Our results show psychometric validity for PFDI-20 questionnaire and are comparable with previous validation studies [8–10]. In our study, PFIQ-7 had some limitations whereas Teleman et al found acceptable psychometric properties in the Swedish version of PFIQ-7 [9]. There was some evidence for a floor effect in our study, although 15–17% of respondents scored the minimum value with each of the subscales, which is considerably less than in the Dutch validation study [10], where the scales of the PFIQ-7 showed floor effects in 44–55% patients, though the summary score did not. Due et al. reported opposite difficulties with the Danish version of PFIQ-7, with a major ceiling effect and lack of items about health-related quality of life [8]. Thus, some but not all the problems with PFIQ-7 in our study may not be due to cultural reasons. It is not clear why the item response rate of PFIQ-7 was low in our study. In future, it may be reasonable to make another linguistic and cultural validation process for the PFIQ-7 to improve the usefulness of the Finnish translation of this instrument. The PISQ-12 showed acceptable psychometric properties as also evaluated in the Swedish study [9]. Limitation of both studies is the relatively small number of sexually active patients (*N* = 25 in reference [9], *N* = 27 in our study). Another limitation of PISQ-12 is that it measures the sexual function only among sexually active heterosexual women. Therefore, another instrument to measure pelvic floor disorders’ impact on sexual activity for both sexually active and inactive women has been published [25]. This IUGA-revised questionnaire (PISQ-IR) [26] has already been translated and validated into five languages [25, 27–30]. In the future, it would be reasonable to conduct PISQ-IR translation and validation processes also in Finnish to assess its validity in the Finnish context.

The multistep translation method was one of the strengths of this study. The existing evidence supports this approach over the more simple translation – back translation process [21]. The translation and linguistic validation process was conducted in accordance with the guidelines for the translation and cultural adaptation of patient-reported outcome (PRO) measures [31]. We used four different translations and a multi-professional team in the translation process. Cognitive debriefing of the translated versions was done to ensure consistent and accurate interpretation and understanding of the questionnaires among respondents. One of the lay persons was bilingual with English as another home language. The study subjects of this multicenter study represent sufficiently different geographical areas and dialects of the Finnish language. The age distribution (mean 64.1 years, median 64 years) in our study represents the typical age of women suffering from symptomatic pelvic prolapse [3].

One limitation of the study was that we did not record the socioeconomic position of the patients. Hence, it may be possible that, for example, patients with higher education were over-presented in the study. Another, but in our opinion minor drawback in a study of this kind is the overall response rate of only 52%. In the Danish study of Due et al. [8], in which the recruiting process was similar to ours, the response rate was 60%. Reasons for the lower response rate may be the lack of personal contact with the subjects when the forms were sent and the fact that there were no reminders.

## Conclusions

In conclusion, the Finnish versions of PFDI-20 and PISQ-12 are reliable, valid and feasible to evaluate the symptoms and the quality of life in women with pelvic floor disorders. Instead, the Finnish version of PFIQ-7 has some limitations and is not usable in its current form. We suggest that PFDI-20 and PISQ-12 should be used as a patient-reported outcome measure in research and clinical practice.

## Additional files

Additional file 1: PFDI-20 in Finnish. (DOCX 114 kb)

Additional file 2: PFIQ-7 in Finnish. (DOCX 100 kb)

Additional file 3: PISQ-12 in Finnish. (DOCX 86 kb)

Additional file 4: Table S1. Item-total correlations for PFIQ-7 and its subscales. (DOCX 14 kb)

Additional file 5: Table S2. Item-total correlations for PFDI-20 and its subscales. (DOCX 15 kb)

Additional file 6: Table S3. Item-total correlations for PISQ-12. (DOCX 12 kb)

## Abbreviations

CRADI: Colo-rectal-anal distress inventory
CRAIQ: Colo-rectal-anal impact questionnaire
HRQOL: Health related quality of life
PFDI: Pelvic floor distress inventory
PFIQ: Pelvic floor impact questionnaire
PISQ: Pelvic organ prolapse/urinary incontinence sexual questionnaire
POP: Pelvic organ prolapse
POPDI: Pelvic organ prolapse distress inventory
POPDI: Pelvic organ prolapse distress inventory
PRO: Patient-reported outcome
UDI: Urinary distress inventory
