# Supplementary material for: Validation of the short forms of the Pelvic Floor Distress Inventory (PFDI-20), Pelvic Floor Impact Questionnaire (PFIQ-7), and Pelvic Organ Prolapse/Urinary Incontinence Sexual Questionnaire (PISQ-12) in Finnish
Source: Health Qual Life Outcomes. 2017 May 2;15:88. doi: 10.1186/s12955-017-0648-2 (PMC5414223; doi:10.1186/s12955-017-0648-2)
Supplement: Supplementary file 1 — PFDI-20 in Finnish. (DOCX 114 kb) [file 12955_2017_648_MOESM1_ESM.docx]

**Lantionpohjavaivojen kartoitus (PFDI-20)**

**Ohjeet:** Kysymysten tarkoituksena on kartoittaa mikäli teillä esiintyy tiettyjä tuntemuksia suolen, virtsarakon tai alapään alueelta, ja kuinka paljon nämä oireet teitä vaivaavat. Vastatkaa kysymyksiin laittamalla rasti sopivaan ruutuun. Vastatessanne kysymyksiin ottakaa huomioon oireenne **viimeisten kolmen kuukauden aikana.**

**POPDI-6**

1. **Onko teillä usein paineen tunnetta alavatsalla?**

☐ Ei ☐ Kyllä

Jos vastasitte kyllä, kuinka paljon se haittaa teitä?

☐ Ei lainkaan ☐ Jonkin verran ☐ Melko paljon ☐ Paljon

1. **Esiintyykö teillä painon tunnetta tai särkyä (jomotusta) alapäässä?**

☐ Ei ☐ Kyllä

Jos vastasitte kyllä, kuinka paljon se haittaa teitä?

☐ Ei lainkaan ☐ Jonkin verran ☐ Melko paljon ☐ Paljon

1. **Esiintyykö teillä pullistuma alapäässä, jonka voitte itse nähdä tai tuntea emättimen ulkosuulla?**

☐ Ei ☐ Kyllä

Jos vastasitte kyllä, kuinka paljon se haittaa teitä?

☐ Ei lainkaan ☐ Jonkin verran ☐ Melko paljon ☐ Paljon

1. **Joudutteko koskaan painamaan emättimestä tai peräaukon läheltä saadaksenne ulostettua?**

☐ Ei ☐ Kyllä

Jos vastasitte kyllä, kuinka paljon se haittaa teitä?

☐ Ei lainkaan ☐ Jonkin verran ☐ Melko paljon ☐ Paljon

1. **Tuntuuko teistä usein siltä, että virtsarakkonne ei tyhjene kokonaan?**

☐ Ei ☐ Kyllä

Jos vastasitte kyllä, kuinka paljon se haittaa teitä?

☐ Ei lainkaan ☐ Jonkin verran ☐ Melko paljon ☐ Paljon

1. **Joudutteko joskus painamaan pullistumaa emättimen sisään aloittaaksenne virtsaamisen tai saadaksenne virtsarakon tyhjenemään?**

☐ Ei ☐ Kyllä

Jos vastasitte kyllä, kuinka paljon se haittaa teitä?

☐ Ei lainkaan ☐ Jonkin verran ☐ Melko paljon ☐ Paljon

**POPDI-6 pisteet x 25=_______**

**CRADI-8**

1. **Joudutteko ponnistelemaan liikaa saadaksenne ulostettua?**

☐ Ei ☐ Kyllä

Jos vastasitte kyllä, kuinka paljon se haittaa teitä?

☐ Ei lainkaan ☐ Jonkin verran ☐ Melko paljon ☐ Paljon

1. **Tuntuuko teistä ulostamisen jälkeen siltä, ettei suoli ole tyhjentynyt kunnolla?**

☐ Ei ☐ Kyllä

Jos vastasitte kyllä, kuinka paljon se haittaa teitä?

☐ Ei lainkaan ☐ Jonkin verran ☐ Melko paljon ☐ Paljon

1. **Onko teillä vaikeuksia pidättää ulostetta , jos uloste on normaalia?**

☐ Ei ☐ Kyllä

Jos vastasitte kyllä, kuinka paljon se haittaa teitä?

☐ Ei lainkaan ☐ Jonkin verran ☐ Melko paljon ☐ Paljon

1. **Onko teillä vaikeuksia pidättää ulostetta, jos uloste on löysää?**

☐ Ei ☐ Kyllä

Jos vastasitte kyllä, kuinka paljon se haittaa teitä?

☐ Ei lainkaan ☐ Jonkin verran ☐ Melko paljon ☐ Paljon

1. **Karkaako teiltä usein kaasu peräsuolesta?**

☐ Ei ☐ Kyllä

Jos vastasitte kyllä, kuinka paljon se haittaa teitä?

☐ Ei lainkaan ☐ Jonkin verran ☐ Melko paljon ☐ Paljon

1. **Onko ulostaminen teille usein kivuliasta?**

☐ Ei ☐ Kyllä

Jos vastasitte kyllä, kuinka paljon se haittaa teitä?

☐ Ei lainkaan ☐ Jonkin verran ☐ Melko paljon ☐ Paljon

1. **Tuleeko teille pakottava ulostamistarve ja kiire vessaan ennen ulostamista?**

☐ Ei ☐ Kyllä

Jos vastasitte kyllä, kuinka paljon se haittaa teitä?

☐ Ei lainkaan ☐ Jonkin verran ☐ Melko paljon ☐ Paljon

1. **Pullistuuko osa peräsuoltanne koskaan ulos peräaukosta ulostamisen aikana tai sen jälkeen?**

☐ Ei ☐ Kyllä

Jos vastasitte kyllä, kuinka paljon se haittaa teitä?

☐ Ei lainkaan ☐ Jonkin verran ☐ Melko paljon ☐ Paljon

**CRADI-8 pisteet x 25=______**

**UDI-6**

1. **Onko teillä tavallisesti tihentynyttä virtsaamistarvetta?**

☐ Ei ☐ Kyllä

Jos vastasitte kyllä, kuinka paljon se haittaa teitä?

☐ Ei lainkaan ☐ Jonkin verran ☐ Melko paljon ☐ Paljon

1. **Karkaako virtsa silloin kun tunnette virtsapakkoa eli hyvin voimakasta virtsaamisen tarvetta?**

☐ Ei ☐ Kyllä

Jos vastasitte kyllä, kuinka paljon se haittaa teitä?

☐ Ei lainkaan ☐ Jonkin verran ☐ Melko paljon ☐ Paljon

1. **Karkaako teiltä tavallisesti virtsaa yskiessä, nauraessa tai aivastaessa?**

☐ Ei ☐ Kyllä

Jos vastasitte kyllä, kuinka paljon se haittaa teitä?

☐ Ei lainkaan ☐ Jonkin verran ☐ Melko paljon ☐ Paljon

1. **Karkaako teiltä tavallisesti pieniä määriä virtsaa (tipoittain)?**

☐ Ei ☐ Kyllä

Jos vastasitte kyllä, kuinka paljon se haittaa teitä?

☐ Ei lainkaan ☐ Jonkin verran ☐ Melko paljon ☐ Paljon

1. **Onko teillä tavallisesti vaikeuksia tyhjentää virtsarakkonne?**

☐ Ei ☐ Kyllä

Jos vastasitte kyllä, kuinka paljon se haittaa teitä?

☐ Ei lainkaan ☐ Jonkin verran ☐ Melko paljon ☐ Paljon

1. **Onko teillä tavallisesti kipua tai epämiellyttävää tunnetta alavatsalla tai alapäässä?**

☐ Ei ☐ Kyllä

Jos vastasitte kyllä, kuinka paljon se haittaa teitä?

☐ Ei lainkaan ☐ Jonkin verran ☐ Melko paljon ☐ Paljon

**UDI-6 pisteet x 25=_____**

Pisteyttäminen: Laske kunkin osion pisteiden keskiarvo (0–4) ja kerro se 25:llä saadaksesi kokonaispistemäärän (asteikolla 0 – 100). Vastaamatta jääneitä kysymyksiä ei huomioida pistelaskussa, vaan keskiarvo lasketaan ainoastaan vastattujen kysymysten pisteistä.

PFDI-20 Pisteytyksen yhteenveto: Laske kaikkien kolmen osion pisteet yhteen saadaksesi kokonaispistemäärän (asteikolla 0 – 100).

**POPDI-6 / CRADI-8 / UDI-6 PFDI-20 PISTEET** _____________

Center for Female Continence PFIQ-7

POTILAAN NIMIKIRJAIMET______PVM._________HENKILÖTUNNUS______________________TUTKIMUSPAIKKA_______

Pre , 3 kk , 6 kk, 12 kk, 24 kk, 36 kk, 60 kk
