# Supplementary material for: Validation of the short forms of the Pelvic Floor Distress Inventory (PFDI-20), Pelvic Floor Impact Questionnaire (PFIQ-7), and Pelvic Organ Prolapse/Urinary Incontinence Sexual Questionnaire (PISQ-12) in Finnish
Source: Health Qual Life Outcomes. 2017 May 2;15:88. doi: 10.1186/s12955-017-0648-2 (PMC5414223; doi:10.1186/s12955-017-0648-2)
Supplement: Supplementary file 2 — PFIQ-7 in Finnish. (DOCX 100 kb) [file 12955_2017_648_MOESM2_ESM.docx]

***Lantionpohjan kunnon merkitys -kysely***

*Täyttöohjeet*: Jotkut naiset kokevat, että rakon, suoliston ja emättimen alueen oireilu vaikuttaa heidän toimiinsa, suhteisiinsa ja tunteisiinsa. Rastittakaa jokaisen kysymyksen kohdalla sellainen vastausvaihtoehto, joka parhaiten kuvaa sitä, kuinka suuri vaikutus rakko-, suolisto-, ja emätinoireilla on ollut teidän toimiinne, suhteisiinne tai tunteisiinne viimeisen kolmen kuukauden aikana. Merkitkää vastauksenne jokaiseen kolmeen sarakkeeseen kaikkien kysymysten kohdalla.

| Kuinka paljon sarakkeisiin merkittyjen ruumiinosien oireilu tavallisesti vaikuttaa | Rakko ja virtsaaminen | Suoli ja peräaukko | Emätin ja lantion alue |
| --- | --- | --- | --- |
| 1. Kykyynne tehdä kotitöitä (esim. ruuanlaitto, pyykinpesu, siivoaminen)? | □ Ei ollenkaan  □ Jonkin verran  □ Melko paljon  □ Paljon | □ Ei ollenkaan  □ Jonkin verran  □ Melko paljon  □ Paljon | □ Ei ollenkaan  □ Jonkin verran  □ Melko paljon  □ Paljon |
| 2. Kykyynne harrastaa liikuntaa, kuten esim. kävelyä, uimista tms.? | □ Ei ollenkaan  □ Jonkin verran  □ Melko paljon  □ Paljon | □ Ei ollenkaan  □ Jonkin verran  □ Melko paljon  □ Paljon | □ Ei ollenkaan  □ Jonkin verran  □ Melko paljon  □ Paljon |
| 3. Kykyynne käydä viihdetilaisuuksissa, kuten esim. elokuvissa tai konserteissa? | □ Ei ollenkaan  □ Jonkin verran  □ Melko paljon  □ Paljon | □ Ei ollenkaan  □ Jonkin verran  □ Melko paljon  □ Paljon | □ Ei ollenkaan  □ Jonkin verran  □ Melko paljon  □ Paljon |
| 4. Kykyynne matkustaa autolla tai bussilla kauemmas kuin 30 minuutin matkan päähän kotoanne? | □ Ei ollenkaan  □ Jonkin verran  □ Melko paljon  □ Paljon | □ Ei ollenkaan  □ Jonkin verran  □ Melko paljon  □ Paljon | □ Ei ollenkaan  □ Jonkin verran  □ Melko paljon  □ Paljon |
| 5. Kykyynne osallistua sosiaaliseen elämään muualla kuin kotonanne? | □ Ei ollenkaan  □ Jonkin verran  □ Melko paljon  □ Paljon | □ Ei ollenkaan  □ Jonkin verran  □ Melko paljon  □ Paljon | □ Ei ollenkaan  □ Jonkin verran  □ Melko paljon  □ Paljon |
| 6. Tunne-elämänne vaihteluihin (esim. jännittäminen ja masennus)? | □ Ei ollenkaan  □ Jonkin verran  □ Melko paljon  □ Paljon | □ Ei ollenkaan  □ Jonkin verran  □ Melko paljon  □ Paljon | □ Ei ollenkaan  □ Jonkin verran  □ Melko paljon  □ Paljon |
| 7. Turhautumisen tunteeseen? | □ Ei ollenkaan  □ Jonkin verran  □ Melko paljon  □ Paljon | □ Ei ollenkaan  □ Jonkin verran  □ Melko paljon  □ Paljon | □ Ei ollenkaan  □ Jonkin verran  □ Melko paljon  □ Paljon |
