# Supplementary material for: Validation of the short forms of the Pelvic Floor Distress Inventory (PFDI-20), Pelvic Floor Impact Questionnaire (PFIQ-7), and Pelvic Organ Prolapse/Urinary Incontinence Sexual Questionnaire (PISQ-12) in Finnish
Source: Health Qual Life Outcomes. 2017 May 2;15:88. doi: 10.1186/s12955-017-0648-2 (PMC5414223; doi:10.1186/s12955-017-0648-2)
Supplement: Supplementary file 3 — PISQ-12 in Finnish. (DOCX 86 kb) [file 12955_2017_648_MOESM3_ESM.docx]

***Lantionpohjan laskeuman /Pidätyskyvyttömyyden vaikutus seksielämään –kyselylomake***

Tämä lomake sisältää kysymyksiä teidän ja partnerinne seksielämästä. Kaikki antamanne vastaukset ovat luottamuksellisia ja niitä käsittelevät ainoastaan lääkärit ymmärtääkseen, mitkä asiat potilaat kokevat tärkeiksi seksielämälleen. Kunkin kysymyksen kohdalla *rastittakaa vastaus, joka parhaiten vastaa omaa kokemustanne*. Vastatessanne kysymyksiin ottakaa huomioon seksielämänne viimeisen kuuden kuukauden ajalta.

**Oletteko tällä hetkellä seksuaalisesti aktiivinen?**

*Rastittakaa sopivin vastausvaihtoehto*

□ Ei, en kykene seksiin (Kiitos vastauksesta, lomaketta ei tarvitse täyttää)

□ En, minulla on liikaa kipuja (Kiitos vastauksesta, lomaketta ei tarvitse täyttää)

□ Ei, en ole halukas (Kiitos vastauksesta, lomaketta ei tarvitse täyttää)

□ En, minulla ei ole partneria (Kiitos vastauksesta, lomaketta ei tarvitse täyttää)

□ En, partnerini ei kykene seksiin (Kiitos vastauksesta, lomaketta ei tarvitse täyttää)

□ Kyllä

**Jos vastasitte EI / EN, lomake on osaltanne täytetty.**

**Jos vastasitte KYLLÄ, jatkakaa vastaamalla seuraaviin 12 kysymykseen (PISQ-12).**

*Kysymykset alkavat seuraavalla sivulla.*

1. **Kuinka usein tunnette sukupuolista halukkuutta?**

Tunne voi käsittää toiveen seksistä, suunnitelmia seksin harrastamisesta, turhautuneisuus seksin puutteen takia, jne.

□ Aina □ Usein □ Joskus □ Harvoin □ En koskaan

1. **Saatteko orgasmin ollessanne yhdynnässä kumppaninne kanssa?**

□ Aina □ Usein □ Joskus □ Harvoin □ En koskaan

1. **Tunnetteko olevanne seksuaalisesti kiihottunut harrastaessanne seksiä kumppaninne kanssa?**

□ Aina □ Usein □ Joskus □ Harvoin □ En koskaan

1. **Oletteko tyytyväinen seksielämäänne ja sen vaihtelevuuteen?**

□ Aina □ Usein □ Joskus □ Harvoin □ En koskaan

1. **Tunnetteko kipua yhdynnän aikana?**

□ Aina □ Usein □ Joskus □ Harvoin □ En koskaan

1. **Onko Teillä usein virtsakarkailua seksin aikana?**

□ Aina □ Usein □ Joskus □ Harvoin □ Ei koskaan

1. **Rajoittaako pelko ulosteen tai virtsan karkailusta seksuaalista aktiivisuuttanne?**

□ Aina □ Usein □ Joskus □ Harvoin □ Ei koskaan

1. **Vältättekö yhdyntää emättimen pullistuman vuoksi (rakon, peräsuolen tai emättimen ulosluiskahtamisen takia)?**

□ Aina □ Usein □ Joskus □ Harvoin □ En koskaan

1. **Kun harrastatte seksiä kumppaninne kanssa, tunnetteko negatiivisia tunteita kuten pelkoa, vastenmielisyyttä, häpeää tai syyllisyyttä?**

□ Aina □ Usein □ Joskus □ Harvoin □ En koskaan

1. **Onko kumppanillanne erektiohäiriö, joka vaikuttaa sukupuolielämäänne?**

□ Aina □ Usein □ Joskus □ Harvoin □ Ei koskaan

1. **Onko kumppanillanne ennenaikaisen siemensyöksyn ongelma, joka haittaa sukupuolielämäänne?**

□ Aina □ Usein □ Joskus □ Harvoin □ Ei koskaan

1. **Kuinka voimakkaita viimeisten kuuden kuukauden aikana tuntemanne orgasmit ovat verrattuna aikaisemmin saamiinne orgasmeihin?**

□ Paljon vähemmän voimakkaita

□ Vähemmän voimakkaita

□ Yhtä voimakkaita

□ Voimakkaampia

□ Paljon voimakkaampia
