# Supplementary material for: Validation of the short forms of the Pelvic Floor Distress Inventory (PFDI-20), Pelvic Floor Impact Questionnaire (PFIQ-7), and Pelvic Organ Prolapse/Urinary Incontinence Sexual Questionnaire (PISQ-12) in Finnish
Source: Health Qual Life Outcomes. 2017 May 2;15:88. doi: 10.1186/s12955-017-0648-2 (PMC5414223; doi:10.1186/s12955-017-0648-2)
Supplement: Supplementary file 4 — Item-total correlations for PFIQ-7 and its subscales. (DOCX 14 kb) [file 12955_2017_648_MOESM4_ESM.docx]

Additional file 1: Table S1 Item-total correlations for PFIQ-7 and its subscales

| PFIQ-7 | *r* | UIQ-7 | *r* | CRAIQ-7 | *r* | POPIQ-7 | R |
| --- | --- | --- | --- | --- | --- | --- | --- |
| CRAIQ-7 Q7 | 0.513 | Q1 | 0.601 | Q6 | 0.568 | Q2 | 0.643 |
| CRAIQ-7 Q6 | 0.536 | Q7 | 0.680 | Q7 | 0.623 | Q6 | 0.667 |
| POPIQ-7 Q2 | 0.566 | Q2 | 0.688 | Q1 | 0.654 | Q1 | 0.704 |
| CRAIQ-7 Q2 | 0.618 | Q6 | 0.707 | Q2 | 0.689 | Q7 | 0.714 |
| UIQ-7 Q6 | 0.638 | Q4 | 0.815 | Q4 | 0.846 | Q5 | 0.849 |
| UIQ-7 Q1 | 0.640 | Q5 | 0.866 | Q5 | 0.893 | Q4 | 0.849 |
| POPIQ-7 Q6 | 0.651 | Q3 | 0.878 | Q3 | 0.901 | Q3 | 0.853 |
| POPIQ-7 Q1 | 0.658 |  |  |  |  |  |  |
| UIQ-7 Q2 | 0.678 |  |  |  |  |  |  |
| POPIQ-7 Q7 | 0.679 |  |  |  |  |  |  |
| CRAIQ-7 Q1 | 0.687 |  |  |  |  |  |  |
| UIQ-7 Q7 | 0.742 |  |  |  |  |  |  |
| UIQ-7 Q4 | 0.780 |  |  |  |  |  |  |
| CRAIQ-7 Q4 | 0.799 |  |  |  |  |  |  |
| UIQ-7 Q3 | 0.801 |  |  |  |  |  |  |
| CRAIQ-7 Q5 | 0.810 |  |  |  |  |  |  |
| CRAIQ-7 Q3 | 0.818 |  |  |  |  |  |  |
| UIQ-7 Q5 | 0.840 |  |  |  |  |  |  |
| POPIQ-7 Q5 | 0.842 |  |  |  |  |  |  |
| POPIQ-7 Q3 | 0.865 |  |  |  |  |  |  |
| POPIQ-7 Q4 | 0.865 |  |  |  |  |  |  |
