# Supplementary material for: Validation of the short forms of the Pelvic Floor Distress Inventory (PFDI-20), Pelvic Floor Impact Questionnaire (PFIQ-7), and Pelvic Organ Prolapse/Urinary Incontinence Sexual Questionnaire (PISQ-12) in Finnish
Source: Health Qual Life Outcomes. 2017 May 2;15:88. doi: 10.1186/s12955-017-0648-2 (PMC5414223; doi:10.1186/s12955-017-0648-2)
Supplement: Supplementary file 5 — Item-total correlations for PFDI-20 and its subscales. (DOCX 15 kb) [file 12955_2017_648_MOESM5_ESM.docx]

Additional file 2: Table S2 Item-total correlations for PFDI-20 and its subscales

| PFDI-20 | *r* | POPDI-6 | *r* | CRADI-8 | *r* | UDI-6 | *R* |
| --- | --- | --- | --- | --- | --- | --- | --- |
| POPDI-6 Q3 | 0.309 | Q3 | 0.309 | Q8 | 0.335 | Q3 | 0.371 |
| POPDI-6 Q3 | 0.310 | Q6 | 0.411 | Q1 | 0.459 | Q4 | 0.382 |
| CRADI-8 Q8 | 0.322 | Q4 | 0.465 | Q7 | 0.502 | Q6 | 0.410 |
| POPDI-6 Q6 | 0.339 | Q5 | 0.490 | Q5 | 0.530 | Q2 | 0.484 |
| UDI-6 Q3 | 0.388 | Q1 | 0.534 | Q3 | 0.535 | Q1 | 0.484 |
| CRADI-8 Q1 | 0.403 | Q2 | 0.579 | Q6 | 0.567 | Q5 | 0.486 |
| POPDI-6 Q6 | 0.411 |  |  | Q2 | 0.597 |  |  |
| UDI-6 Q4 | 0.429 |  |  | Q4 | 0.611 |  |  |
| UDI-6 Q1 | 0.438 |  |  |  |  |  |  |
| CRADI-8 Q6 | 0.460 |  |  |  |  |  |  |
| CRADI-8 Q7 | 0.463 |  |  |  |  |  |  |
| CRADI-8 Q5 | 0.464 |  |  |  |  |  |  |
| POPDI-6 Q4 | 0.465 |  |  |  |  |  |  |
| UDI-6 Q6 | 0.470 |  |  |  |  |  |  |
| UDI-6 Q5 | 0.475 |  |  |  |  |  |  |
| POPDI-6 Q5 | 0.490 |  |  |  |  |  |  |
| POPDI-6 Q1 | 0.534 |  |  |  |  |  |  |
| POPDI-6 Q4 | 0.549 |  |  |  |  |  |  |
| POPDI-6 Q2 | 0.555 |  |  |  |  |  |  |
| CRADI-8 Q2 | 0.555 |  |  |  |  |  |  |
| POPDI-6 Q5 | 0.576 |  |  |  |  |  |  |
| POPDI-6 Q2 | 0.579 |  |  |  |  |  |  |
| UDI-6 Q2 | 0.598 |  |  |  |  |  |  |
| POPDI-6 Q1 | 0.606 |  |  |  |  |  |  |
| CRADI-8 Q4 | 0.615 |  |  |  |  |  |  |
| CRADI-8 Q3 | 0.639 |  |  |  |  |  |  |
