# Supplementary material for: Validation of the short forms of the Pelvic Floor Distress Inventory (PFDI-20), Pelvic Floor Impact Questionnaire (PFIQ-7), and Pelvic Organ Prolapse/Urinary Incontinence Sexual Questionnaire (PISQ-12) in Finnish
Source: Health Qual Life Outcomes. 2017 May 2;15:88. doi: 10.1186/s12955-017-0648-2 (PMC5414223; doi:10.1186/s12955-017-0648-2)
Supplement: Supplementary file 6 — Item-total correlations for PISQ-12. (DOCX 12 kb) [file 12955_2017_648_MOESM6_ESM.docx]

Additional file 3: Table S3 Item-total correlations for PISQ-12

| PISQ-12 | *R* |
| --- | --- |
| Q6 | 0.138 |
| Q11 | 0.216 |
| Q10 | 0.297 |
| Q2 | 0.380 |
| Q7 | 0.446 |
| Q4 | 0.551 |
| Q9 | 0.601 |
| Q5 | 0.619 |
| Q3 | 0.646 |
| Q8 | 0.684 |
| Q12 | 0.687 |
| Q1 | 0.711 |
